# Toward Consistent Canopy Characterization From Low‐Cost UAV Imagery

**DOI:** 10.1002/ece3.74260

**Published:** 2026-09-02

**Authors:** Antoine Plumacker, Bhely Angoboy Ilondea, Nicolas Barbier, Thibauld Collet, Hugo de Lame, Pauline Depoortere, Alexia Favaro, Adeline Fayolle, Marjane Kaddouri, Carmel Mounziegou‐Mounziegou, Pierre Ploton, Zoé Rousseau, Edmond Saint Denis, Paul Tresson, Hadrien Tulet, Arthur Vander Linden, Jean‐François Bastin

**Affiliations:** ^1^ TERRA Teaching and Research Centre, Gembloux Agro‐Bio Tech Université de Liège Gembloux Belgium; ^2^ Institut National pour l'Étude et la Recherche Agronomiques (INERA) Kinshasa Democratic Republic of the Congo; ^3^ Université Pédagogique Nationale Kinshasa Democratic Republic of the Congo; ^4^ AMAP, IRD, CNRS, INRAE, CIRAD Univ Montpellier Montpellier France; ^5^ Forêts et Sociétés, CIRAD Montpellier Univ Montpellier Montpellier France; ^6^ IGN Saint‐Mandé France

## Abstract

Monitoring tropical forest structure at landscape scale requires cost‐effective methods capable of bridging the gap between field inventories and satellite remote sensing products. However, the diversity of available individual tree crown (ITC) segmentation algorithms raises questions about the consistency and reliability of derived structural metrics—a critical issue for any ecological application relying on these outputs. We evaluated three ITC segmentation algorithms—Detectree2, SAM, and the hybrid Detectree2SAM (D2S)—applied to very high‐resolution RGB orthomosaics (5 cm resolution) acquired over ~200 ha of the Luki Biosphere Reserve (Democratic Republic of Congo) using a low‐cost drone. Algorithms were validated at the individual scale against a photointerpretation reference of 1882 manually delineated crowns and at the plot scale against a field inventory of 360 trees across 18 plots. At the individual scale, IoU‐based F1 scores ranged from 0.57 (D2S) to 0.67 (SAM), revealing clear precision–recall trade‐offs. At the plot scale, D2S provided the most accurate crown area estimates (RMSD = 26%–29%), while SAM best reproduced canopy density (RMSD = 22%). All three algorithms systematically overestimated aggregated metrics; a linear correction substantially reduced prediction errors and improved cross‐algorithm convergence, yielding consistent estimates—median crown area of the 20 largest trees (~148 m^2^), total crown area (~3850 m^2^ per plot), and canopy density (~80 ind/ha). Agreement maps revealed systematic spatial divergences, including edge artifacts, SAM's tendency to underestimate canopy density, and localized overestimation of crown areas in disturbed stands. Raw ITC outputs carry substantial systematic biases requiring explicit field‐calibrated correction before ecological use. We propose a three‐step operational pipeline—(i) plot‐scale field validation, (ii) per‐algorithm linear bias correction, and (iii) landscape‐scale aggregation—that reduces inter‐algorithm divergence and delivers consistent, ecologically interpretable structural estimates from low‐cost UAV imagery.

## Graphical abstract

We show that UAV‐based crown segmentation can reliably characterize dominant canopy trees in a structurally complex tropical forest. Focusing on these key individuals improves the assessment of forest structure and supports ecological monitoring at multiple scales.

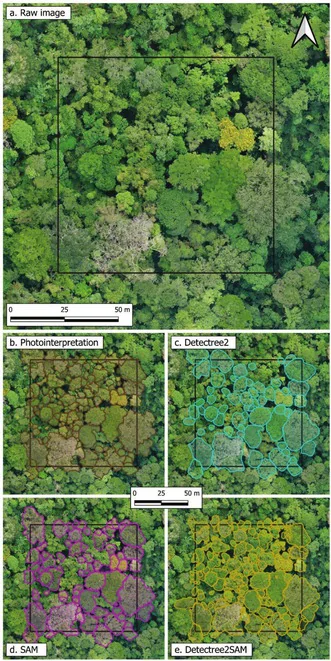

## 1 Introduction

Understanding the role of tropical forests in global change requires monitoring their structure and dynamics with approaches that can handle their high complexity, spatial heterogeneity, and rapid changes. Such monitoring is crucial to assess their sensitivity to climatic variability (e.g., drought, extreme temperatures) and anthropogenic pressures (e.g., deforestation and degradation), as well as to understand their role in climate regulation and biodiversity conservation (Saxe et al. 2001; Zhou et al. 2014).

Tropical forests are species‐rich and structurally complex (multi‐layered) ecosystems. A set of forest attributes is commonly used in cross‐site comparisons (Poorter et al. 2015; Sullivan et al. 2020). While these attributes can be measured through conventional field inventories, such methods remain costly and labor‐intensive (Asner et al. 2015; Berenguer et al. 2014; Labrière et al. 2023). As a result, existing field inventories in Central Africa are generally insufficient to support effective monitoring and understanding of these ecosystems. According to the ForestPlots.net synthesis, about 62% of permanent tropical forest plots are located in South America, 26% in Africa, and 11% in Asia (ForestPlots.net et al. 2021; Lewis et al. 2013), a distribution that partly reflects differences in forest extent across regions. Alternative approaches are therefore critically needed to support data collection, representativeness, and long‐term monitoring. One promising avenue is the supersite framework, which promotes coordinated efforts toward a limited number of well‐instrumented forest sites worldwide (≈200 candidates have been identified; Labrière et al. 2023). These supersites aim to establish a calibration and validation reference dataset for satellite observation products and in particular for the ESA Copernicus BIOMASS mission, providing necessary long‐term, high‐quality measurements of forest structure, dynamics, and carbon stocks (Chave et al. 2021, 2019; Labrière et al. 2023, 2018). In this regard, several scientific developments offer promising perspectives to reduce the cost of field inventories, expand the spatial coverage, and improve our global understanding of tropical forest ecology (de Almeida et al. 2025; Diez et al. 2021; Fassnacht et al. 2024).

Remote sensing is arguably presented as the best answer to expand spatial coverage with a specific focus on forest canopy crowns. While some approaches using very high‐resolution spaceborne data have provided promising perspectives to assess structural attributes in forest ecosystem (Barbier et al. 2010; Bastin et al. 2014; Ploton et al. 2012), new opportunities arise with the rapidly increasing accessibility to airborne drone technology—both regarding image acquisition and image processing. The key advantage of drones over satellites goes beyond spatial resolution: they provide full autonomy in both spatial and temporal data collection, enabling descriptions of forest ecosystems over several hundreds of hectares, whereas censusing a single hectare typically requires 13 person‐days (Phillips et al. 2021). Yet drone‐based remote sensing remains complementary to field inventories, as canopy imagery is inherently limited to the uppermost forest layer.

Although airborne remote sensing from mid‐range commercial drones mainly captures the top of canopy, these canopy trees have been established as key indicators of the structural, compositional, and functional attributes of tropical forest ecosystems, as the dominant individuals drive most of the processes occurring below the canopy (Bastin et al. 2018, 2015; Grime 1998; Lutz et al. 2018; Meyer et al. 2018; Slik et al. 2013; Stegen et al. 2011). Notably, measuring only about the largest 5% of individuals typically reported in field inventories, that is, the 20 largest individuals per hectare in tropical Africa, enables prediction of variations in forest aboveground biomass, basal area, wood density, or even the species richness with an error below 10% (Bastin et al. 2018, 2015; Poulsen et al. 2020; Sillett et al. 2019). Beyond their role as proxies of forest structural attributes, canopy trees may also reflect ecological strategies through their crown architecture. Their shape and size may capture functional strategies of light acquisition, competition, growth, and reproduction (Ehbrecht et al. 2021; Halle et al. 2012). Crown area is a strong predictor of tree DBH and height due to allometric relationships (Blanchard et al. 2016; Jucker et al. 2022; Ploton et al. 2016). Yet, despite this potential, few studies have explicitly linked crown‐level metrics to plot‐scale structural attributes such as canopy height or basal area, although relationships with aboveground biomass density (AGBD) have been established using canopy metrics from LiDAR‐based surveys (Duncanson et al. 2022; Labrière et al. 2018). Exploring these relationships could therefore provide a new pathway for characterizing forest structure from canopy crown measurements. Monitoring canopy crowns could consequently offer a cost‐effective opportunity to assess these attributes on a broader scale.

Tree canopy crowns can be segmented from very high‐resolution imagery using either manual or automated approaches. Such segmentation allows direct measurement of crown‐level structural metrics (e.g., crown area, crown shape complexity), often with accuracy equal to or greater than traditional ground‐based methods (Clark and Kellner 2012; Freudenberg et al. 2022; Li et al. 2023; Speckenwirth et al. 2024). It also provides a way to reduce biases typically encountered when using indirect indicators, such as vegetation indices, to model forest attributes. Automated tree crown segmentation is a sub‐topic of object segmentation, which has been rapidly evolving over the past decade. Convolutional neural networks (CNN) first enabled major advances in image segmentation, before the emergence of transformer‐based architectures that demonstrated strong performance for vision tasks (Dosovitskiy et al. 2021; Vaswani et al. 2017). More recently, self‐supervised learning (SSL) approaches have further improved segmentation performance, particularly when combined with vision transformers (Caron et al. 2021). These developments trickled down to forest remote sensing, where automated tree crown segmentation has benefited from increasingly accurate and scalable methods (Bosch 2020; Firoze et al. 2023; Ke and Quackenbush 2011).

Currently, the Detectree2 algorithm is the state of the art for tree crown segmentation based on aerial RGB images (Ball et al. 2023). This algorithm is based on the Mask R‐CNN deep learning framework (He et al. 2017), designed to accurately delineate tree crowns from aerial RGB images. First trained using 3797 manually annotated tree crowns from four locations across three tropical forest sites—Sepilok Forest Reserve (East and West) and Danum Valley Conservation Area in Sabah, Malaysia, and Paracou Field Station in French Guiana—the model delineated 65,786 tree crowns over an area of 14 km^2^ in one application, demonstrating good performance in complex environments. Although it primarily focused on the tropical forests of Borneo, Malaysia, and French Guiana, the authors highlighted the potential for similar applications in Central African forests. Besides forest‐specific applications, the Segment Anything Model (SAM—Kirillov et al. 2023) is the most widely adopted foundation model for zero‐shot instance segmentation, extensively benchmarked across computer vision tasks, making it a natural reference point for evaluating segmentation approaches in remote sensing (Chen et al. 2025; Osco et al. 2023). SAM was trained for zero‐shot instance segmentation, meaning it produces segmentation masks without additional training. This algorithm relies on a vision transformer (ViT) architecture trained in self‐supervised learning (SSL) as an encoder to map the input image into a feature space. A decoder then leverages this representation to generate segmentation masks of objects present in the image, with or without user‐defined prompts to guide the process. However, its application to geospatial data such as drone images requires specific adaptations (Osco et al. 2023; Wu and Osco 2023).

To bridge the gap between field inventories and wall‐to‐wall satellite‐based products, the present study proposes and validates an operational framework for consistent multi‐scale characterization of forest structure from low‐cost UAV imagery. Applied to the heterogeneous tropical forests of the Luki Biosphere Reserve (Democratic Republic of Congo), we evaluate three ITC segmentation algorithms—Detectree2, SAM, and a novel hybrid approach (Detectree2SAM)—and show that, despite substantial algorithm‐specific biases in raw outputs, a simple field‐calibrated correction pipeline, particularly when focusing on the 20 largest crowns per hectare, can substantially reduce inter‐algorithm divergence and yield ecologically interpretable structural estimates at landscape scale. Rather than treating algorithmic comparison as an end in itself, we position this three‐step pipeline—field validation, bias correction, and landscape aggregation—as a transferable and reproducible approach for tropical forest structural monitoring using affordable drone technology.

We specifically addressed the following research questions: (1) How accurately can Detectree2, SAM, and their combination delineate individual tree crowns when evaluated against a manually photo‐interpreted reference dataset of 1882 trees? (2) To what extent can these approaches characterize plot‐level forest structure—specifically the diversity of crown sizes among the 20 largest canopy trees (median and summed crown area, compared with a field‐based inventory) and tree density (compared with a photo‐interpreted dataset)? (3) How do differences among algorithms manifest first at the individual scale (crown area and shape metrics), and then at the plot scale through the median and summed area of the 20 largest crowns as well as tree density when these indicators are observed across the entire study landscape?

## 2 Materials and Methods

### 2.1 Study Site

**FIGURE 1:**
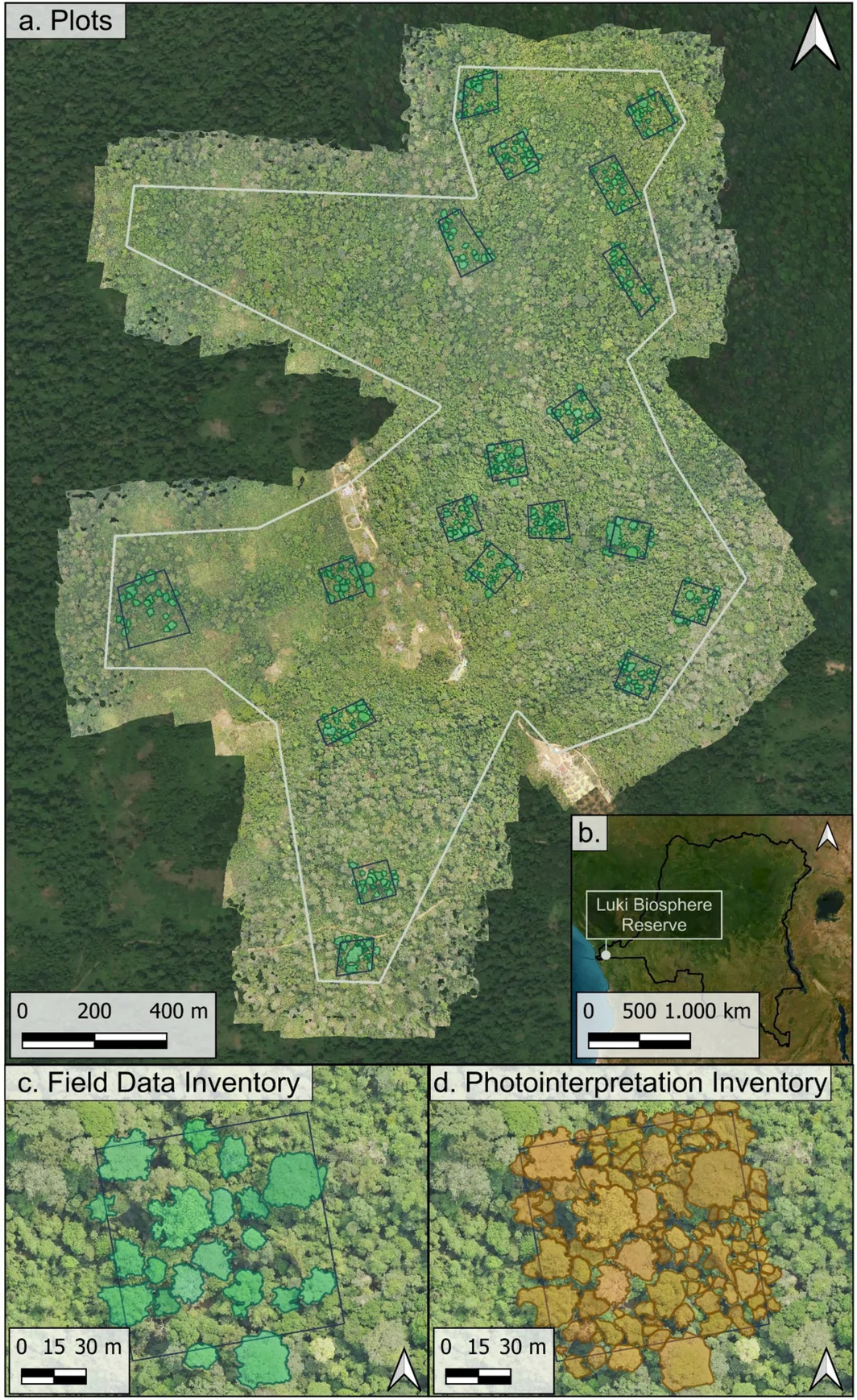
Study site in the Luki MAB Reserve. (a) Orthoimage covering 200 ha around the base camp of the Luki Biosphere Reserve, showing the 19 plots with field‐based crown delineation. (b) Geolocation of the Luki Biosphere Reserve in the Democratic Republic of Congo. (c) Close‐up of a plot with field‐based crown delineation. (d) Close‐up of a plot with crown delineation based on photo‐interpretation.

The study area lies within the Guineo‐Congolian rainforest complex, a major tropical forest domain in Central Africa. In its south‐western portion, the Mayombe forest block is characterized by semi‐deciduous forest elements and marked topographical relief. Within this block, the Luki Biosphere Reserve (Democratic Republic of Congo—5.621° S, 13.099° E—Figure 1b) protects one of the region's better preserved forest patches. The phenological park of Nkula is one of the long‐term monitoring sites established within Luki (Angoboy Ilondea et al. 2019; Couralet 2013). The study area covers ~200 ha within the Nkula phenological park of the Luki Biosphere Reserve and encompasses the 19 plots (~1 ha each) distributed across the study area for forest monitoring purposes (Figure 1a).

The climate is marked by an extended rainy season from October to May (occasionally interrupted by a short dry spell between December and February), followed by a primary dry period from June to September. Temperatures range between ~21°C and 27°C depending on season (Cizungu et al. 2021).

The topography is strongly dissected: elevations range from 150 m to 370 m above sea level, and slopes average ~16.5°, with some local maxima reaching ~70°.

Long‐term phenological data from the Luki Reserve highlight a strong seasonality in reproductive events, with a pronounced annual pattern in flowering and fruiting—flowering often peaking between December and February (the short dry season) while some reproductive activity continues year‐round at the community level (Angoboy Ilondea et al. 2019).

### 2.2 Inventories

We established two dedicated drone‐based inventory datasets to test and validate the models used in this study. The first, called the Field Data Inventory (Figure 1c), was designed to evaluate the prediction of structural attributes derived from segmentation outputs at both the individual tree and plot levels. It relies on field surveys combining dendrometric measurements with geolocation and synchronized crown delineation, conducted directly in the field on an RGB orthomosaic displayed on a tablet. This inventory covers 18 of the 19 plots. The second, referred to as the Photointerpretation Inventory (Figure 1d), was designed to assess the detection accuracy of automatic crown segmentation. It is based on a detailed manual photointerpretation of canopy crowns within 19 plots distributed across the study area (Figure 1a), using a very high‐resolution RGB orthomosaic acquired by drone. Thus, the Field Data Inventory includes 360 trees measured in 18 plots (one plot was excluded due to georeferencing issues), while the Photointerpretation Inventory comprises 1882 manually delineated crowns across 19 plots.

#### 2.2.1 UAV Data Acquisition

Both inventories are based on very high‐resolution RGB orthomosaics acquired using a DJI Mavic 2 Pro drone, equipped with a 1‐in. CMOS sensor (20 MP, 77° FOV, 28 mm equivalent focal length). Flights were performed in a lawnmower pattern—a series of parallel flight lines flown in alternating directions to ensure complete coverage of the study area—using Dronelink (2024) at an altitude of 200 m, a speed of 10 m/s, with 75% forward and side overlap, and nadir camera angle. Terrain following was not enabled. Six flights were conducted around solar noon for a total of approximately 72 min of flight time, under sunny conditions with occasional cloud cover. Orthomosaics were generated in Agisoft Metashape 2.0 (Agisoft 2025) with a ground resolution of 5 cm, using high accuracy photo alignment, high quality depth map generation, and automatic color calibration. Sun sensor correction was not available.

#### 2.2.2 Field Data Inventory (360 Trees on 18 Plots)

Based on this imagery, 18 plots were delineated within the study area. In each plot, the 20 largest trees—defined here as those with the largest crown areas—were identified from an initial photointerpretation‐based delineation. Their diameter at breast height (DBH) was then measured in the field using a measuring tape between 2 and 24 April 2023, during the rainy season. At the same time, tree crowns were checked in the field and, when necessary, manually refined directly on the drone orthomosaic using QField on a Xiaomi Redmi Note 11 (Figure S1).

#### 2.2.3 Photointerpretation Inventory (1882 Trees on 19 Plots)

To assess and compare the different ITC algorithms used and developed in this study, we produced a complementary inventory based entirely on manual photointerpretation, specifically designed to calculate true positives, false positives, true negatives, and false negatives—key indicators for computing Recall and F1 scores, which are commonly used in the scientific literature to evaluate the quality of automated object segmentation. A total of 1882 tree crowns were manually delineated across the 19 plots. It should be noted that photointerpretation of individual crowns in dense tropical forest is inherently challenging, particularly when separating adjacent individuals, including those belonging to the same species. This inventory should therefore be considered a human benchmark rather than an absolute ground truth. This is further supported by a spatial comparison against field‐delineated crowns, which confirmed a generally good agreement between the two delineation approaches (Figure S2).

To improve the accuracy of this delineation, we used two very high‐resolution RGB images each covering the same 200‐ha area. The first image, acquired on May 4, 2023, served as the primary reference for crown delineation in QGIS 3.32.3 and as the input for all subsequent automatic segmentation and landscape‐scale analyses, although it does not coincide with the date of the field inventory due to logistical constraints. The second image, acquired on November 1, 2023, was only used as a visual aid to refine ambiguous crown boundaries during manual photointerpretation, by leveraging seasonal spectral contrasts between adjacent individuals (Figure S3).

### 2.3 Automatic ITC Segmentation Methods

Two ITC algorithms applicable to optical RGB data were tested, and a third approach was developed by combining them to leverage the strengths of each. All automatic segmentation analyses were implemented in Python 3.9.16 using the following key libraries: Detectron2 (Wu et al. 2019) for Detectree2, Segment Anything Model (Kirillov et al. 2023) for SAM, alongside PyTorch 2.1.0, Rasterio 1.4.3, GeoPandas 0.13.0, and Shapely 2.1.1 for geospatial processing. Post‐processing and statistical analyses were performed in R 4.5.1. Analyses were conducted on a Lenovo ThinkBook 16p Gen 2 laptop equipped with an AMD Ryzen 75800H CPU (3.20 GHz), 40 GB RAM, and an NVIDIA GeForce RTX 3060 GPU (6 GB VRAM, CUDA 12.2).

#### 2.3.1 Detectree2

We used Detectree2 (fine‐tuned on UAV data from Paracou—French Guiana), which is based on the Detectron2 implementation of Mask R‐CNN, to detect and segment individual tree crowns under tropical forest conditions (Ball et al. 2023; He et al. 2017). The pre‐trained model was used without changing the default hyperparameters. The orthomosaic was processed in 45 × 45 m tiles with a 20 m overlap buffer at a tile resolution of 1024 pixels, corresponding to a ground sampling distance of ~4.4 cm/pixel, slightly different from the native orthomosaic resolution of 5.0 cm/pixel. Predicted crowns were post‐processed by removing duplicate detections with an IoU > 0.4, filtering out low‐confidence detections (score < 0.2), and discarding crowns smaller than 2 m^2^. The resulting crowns were simplified to reduce geometry complexity and exported as georeferenced shapefiles (Figure S4).

#### 2.3.2 SAM

The Segment Anything Model (SAM) was the second algorithm used (Kirillov et al. 2023). SAM is a computer vision model that was not originally designed for geospatial applications, requiring specific adaptations (Osco et al. 2023; Wu and Osco 2023). The process began with the definition of tiling parameters: tiles of 40 × 40 m with a 60 m overlap buffer, larger than for Detectree2, to mitigate edge artifacts inherent to SAM's prompt‐less segmentation, where crowns straddling tile boundaries tend to be inconsistently segmented. Tile size was selected based on empirical testing to optimize segmentation performance. This was followed by a prompt‐less segmentation and a projection of the resulting segments into UTM coordinates to identify the detected crown positions.

#### 2.3.3 Detectree2SAM (D2S)

**FIGURE 2:**
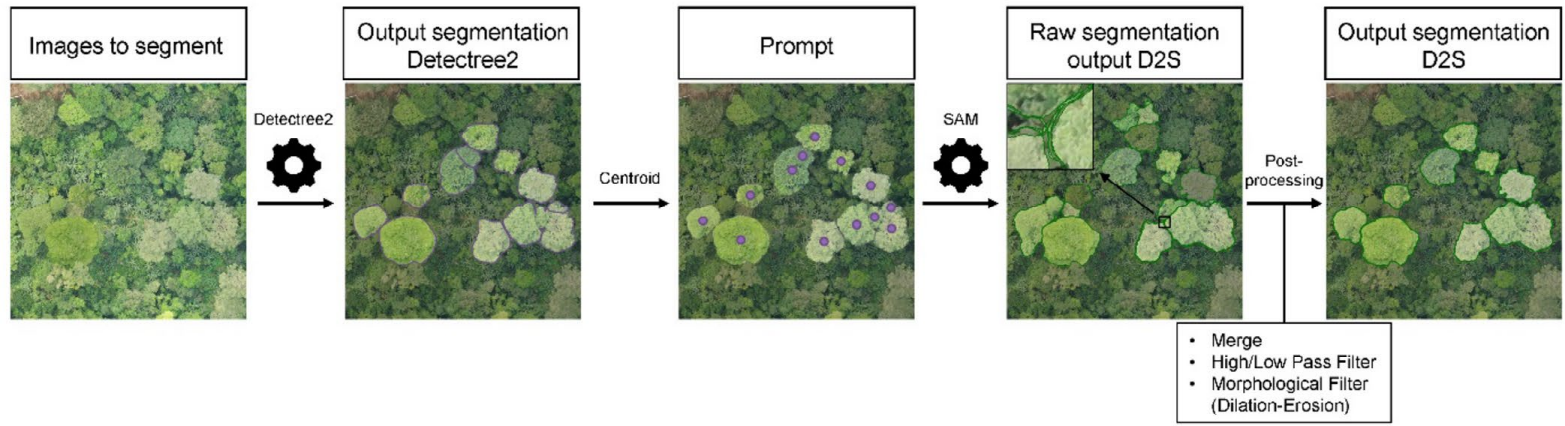
Workflow for the Detectree2SAM (D2S) crown segmentation approach, using Detectree2 centroids as prompts for the SAM model. The segmentation is followed by a post‐processing step including merging, size‐based filtering (minimum/maximum), and morphological operations.

We developed a hybrid segmentation workflow, named Detectree2SAM (D2S), which sequentially combines the strengths of both Detectree2 and SAM. The process starts with the detection of tree centroids using Detectree2. The centroids of predicted polygons with a confidence score greater than 0.2 are used as spatial prompts to guide SAM in crown segmentation, focusing the algorithm on pre‐identified tree locations. Since SAM may produce several overlapping segments for a single object, we applied a post‐processing step to identify unique crowns. This includes merging and filtering segments using area thresholds: a minimum crown area of 2 m^2^ and a maximum of 2500 m^2^, set to exceed the largest crown recorded in the Luki landscape (~2200 m^2^). Additionally, we applied a morphological filter combining dilation and erosion (50 cm), selected empirically, to reduce noise—defined as segmentation artifacts or inaccuracies due to model inconsistencies (Figure 2).

### 2.4 Validation of Crowns at the Individual Scale

#### 2.4.1 Validation of ITC Segmentations

The validation of the Detectree2, SAM, and Detectree2SAM algorithms was performed by comparing model outputs to the photointerpretation inventory, which, unlike the field dataset, aims to cover all visible canopy trees within a plot, with the limitations inherent to this approach acknowledged. Two validation methods were used to assess the accuracy of the automatic segmentations compared to manual segmentations. The first method relies on a pixel‐by‐pixel comparison. The segmented polygons and ground truth were rasterized and then compared using confusion matrices. This allowed for the assessment of the overall accuracy of the automatic segmentations. The second method is based on the Intersection over Union (IoU) metric. A segmentation is considered correct if the IoU is greater than or equal to 50% (Everingham et al. 2010). This threshold is used to determine True Positives (TP), False Positives (FP), and False Negatives (FN), from which precision, recall, and F1 scores are calculated. These metrics provide a more detailed evaluation of the models' performance. For each validation method, a 95% confidence interval was estimated by bootstrap resampling (1000 iterations), randomly resampling individual crown IoU values with replacement at each iteration and computing the 2.5th and 97.5th percentiles of the resulting distribution of F1, precision, and recall scores.

$$\text{Recall}=\frac{\mathrm{TP}}{\mathrm{TP}+\mathrm{FN}}\;\text{Precision}=\frac{\mathrm{TP}}{\mathrm{TP}+\mathrm{FP}}\;F1\;\text{score}=2\times \frac{\text{Precision}\times \text{Recall}}{\text{Precision}+\text{Recall}}$$

### 2.5 Validation of Canopy Structural Attributes at the Plot Scale

At plot scale, several attributes were extracted to characterize canopy structure from both automated segmentations and manual crown inventories. First, metrics related to the 20 largest crowns at the plot scale were evaluated by comparing the median and sum values of crown area between the automated segmentations and those from the field dataset. Similarly, the number of canopy crown individuals estimated per hectare by the ITC algorithms was compared with the photo‐interpreted dataset. The absolute and relative Root Mean Square Deviation (RMSD & RMSD%) were calculated for each of these metrics. Linear models were fitted between field (X) and independent ITC outputs (Y) to assess ITC model biases. If the bias appeared systematic, we derived correction factors from model coefficients, that is, intercept and slope. The RMSD before and after correction, and the relative gain in prediction accuracy (Gain%), were reported for each algorithm and metric.

At landscape scale, tree crown attributes were mapped at a 10‐m pixel resolution, with each pixel value computed by aggregating individual crown metrics within a 1‐ha window centered on that pixel. The linear correction factors derived at plot scale were propagated to all landscape‐scale estimates to produce bias‐adjusted canopy attribute maps. These maps were produced using the algorithm that showed the best agreement with the manual reference datasets, as determined by the lowest RMSD. Spatial differences between algorithms were also visualized by mapping the deviation from the mean of the three segmentation approaches.

Additional attributes, calculated at the individual‐tree scale, were also extracted across the entire landscape: the area of each crown, to estimate the two‐dimensional projected surface covered by individual trees, as well as a shape complexity index for each segmented crown.

$$\frac{2*\sqrt{\pi *\text{area}}}{\text{perimeter}}$$

This index, ranging from 0 to 1, describes crown morphology: a value of 1 corresponds to a perfect circle, values between 0.5 and 1 indicate a slightly irregular shape, while values below 0.5 correspond to increasingly complex, elongated, or irregular crown shapes (Figure S5).

Finally, these attributes were compared among algorithms through a descriptive analysis using density functions.

## 3 Results

### 3.1 Crown Validation at the Individual Scale

**FIGURE 3:**
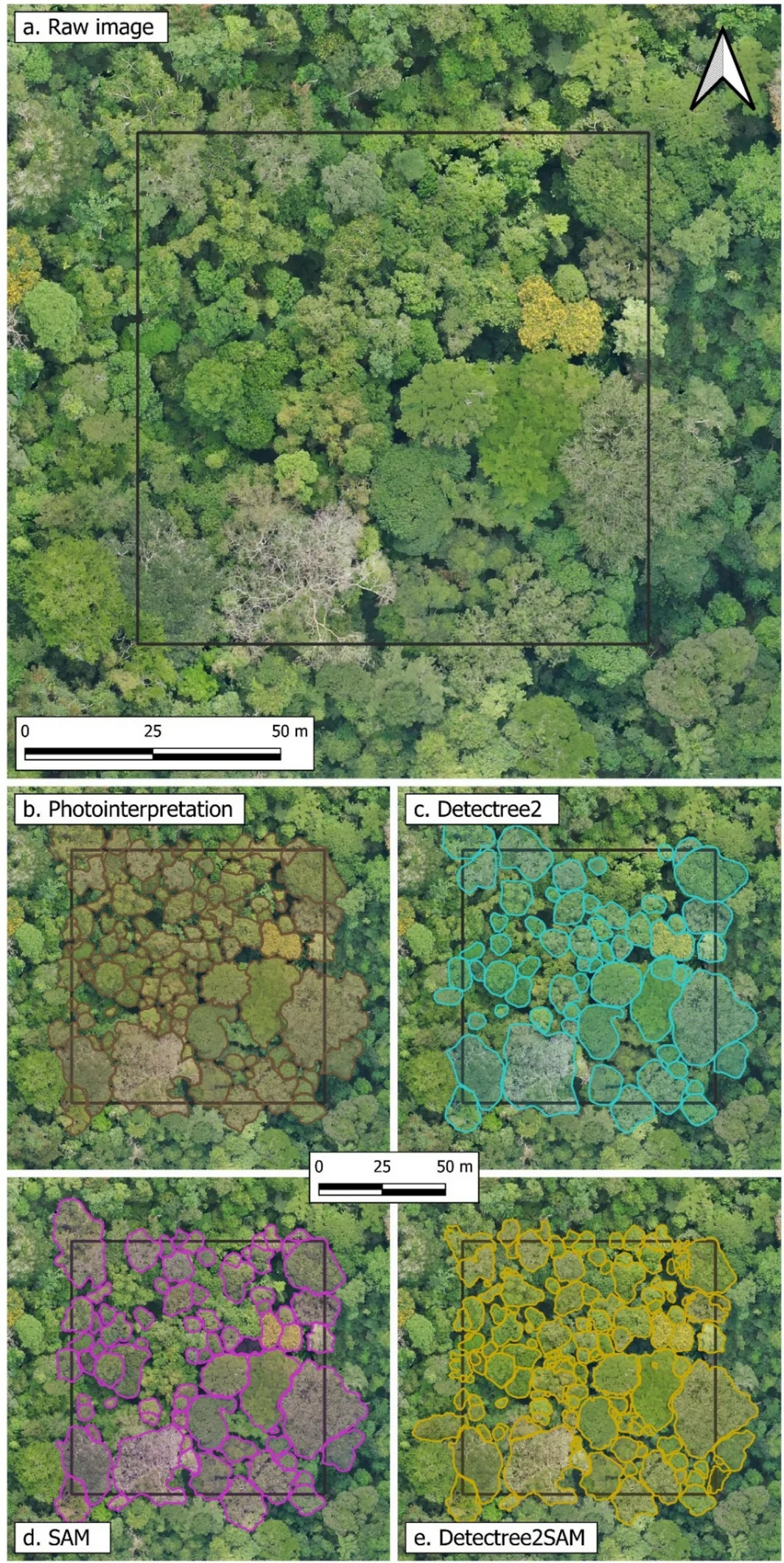
Example outputs of the three ITC algorithms on: (a) 1 ha representative of the Luki Biosphere Reserve site, with (b) photointerpretation, (c) Detectree2, (d) SAM, and (e) Detectree2SAM.

The performance of the three ITC algorithms (Detectree2, SAM, and Detectree2SAM) (Figure 3) was evaluated using two approaches: pixel‐based validation (Table 1) and intersection over union (IoU) validation (Table 1). These methods allow comparison of the algorithms' detections with a reference dataset segmented through photointerpretation. Across the study area, Detectree2 segmented 8684 crowns, SAM segmented 13,955 crowns, and D2S segmented 23,495 crowns.

**TABLE 1:** Pixel‐based and Intersection over Union (IoU)–based validation results of individual tree crown (ITC) segmentation methods compared to the photo‐interpreted reference dataset.

Validation type	Method	Recall (95% CI)	Precision (95% CI)	F1 (95% CI)
Pixel‐based validation	Detectree2	**0.8964** (0.8963–0.8966)	**0.7407** (0.7405–0.7409)	**0.8112** (0.8110–0.8113)
	SAM	**0.8221** (0.8219–0.8223)	**0.8408** (0.8406–0.8410)	**0.8313** (0.8312–0.8315)
	Detectree2SAM	**0.8836** (0.8835–0.8838)	**0.8029** (0.8027–0.8031)	**0.8413** (0.8412–0.8415)
IoU‐based validation	Detectree2	**0.7452** (0.7287–0.7610)	**0.5471** (0.5271–0.5660)	**0.6309** (0.6140–0.6465)
	SAM	**0.6529** (0.6380–0.6681)	**0.6876** (0.6654–0.7085)	**0.6697** (0.6534–0.6850)
	Detectree2SAM	**0.7417** (0.7245–0.7585)	**0.4645** (0.4466–0.4819)	**0.5712** (0.5543–0.5874)

*Note:* Bold values indicate mean performance estimates; values in parentheses indicate 95% bootstrap confidence intervals.

For the pixel‐based validation (Table 1), Detectree2SAM achieved the highest F1 score (0.84), with a recall of 0.88 and a precision of 0.80. Detectree2 showed a similar recall (0.90) but a lower precision (0.74), resulting in an F1 score of 0.81. SAM reached an F1 score of 0.83, with recall (0.82) and precision (0.84) values that were close to each other.

In the IoU‐based validation, F1 scores were generally lower. SAM achieved the highest IoU‐based F1 score among the three algorithms (0.67), indicating that approximately two thirds of the reference crowns were correctly detected with acceptable boundary overlap. Detectree2 showed higher recall (0.75)—detecting three quarters of the reference crowns—but lower precision (0.55) and F1 score (0.63). Detectree2SAM had a recall of 0.74 but the lowest precision (0.47), with an F1 score of 0.57.

The results reveal trade‐offs among the algorithms that vary with the validation method: Detectree2 and Detectree2SAM achieve higher recall, whereas SAM provides higher precision.

### 3.2 Quantitative Comparison of Derived Attributes at Plot Scale

**FIGURE 4:**
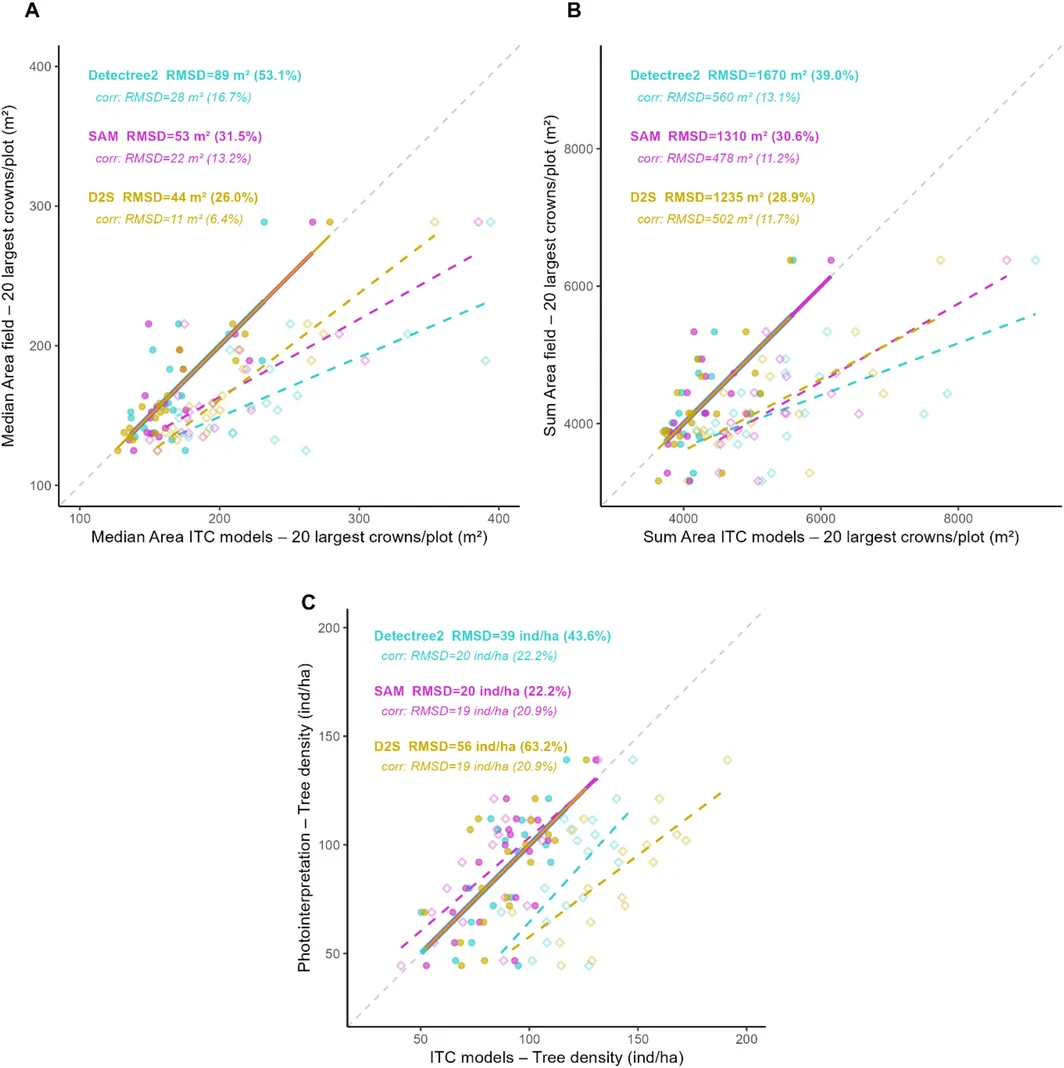
Predictive performance of ITC crown segmentation models at the plot scale (Detectree2, SAM, Detectree2SAM). (A) Median crown area for the 20 largest tree crowns per plot, (B) Sum of crown areas for the 20 largest tree crowns per plot, and (C) Tree density (number of trees per plot). Each point represents a plot and the dashed gray line indicates the 1:1 relationship. Open diamonds and dashed colored regression lines show raw (uncorrected) predictions; filled circles and solid colored regression lines show linearly corrected predictions. Colors indicate algorithms (Cyan = Detectree2, Magenta = SAM, Yellow = Detectree2SAM). Root mean square deviations (RMSD, absolute and relative) are reported before and after linear correction (*corr*:) for each algorithm.

The predictive performance of the three ITC algorithms was evaluated against field measurements considered as reference for two key structural metrics: the median area of the 20 largest crowns per hectare and the total area of the 20 largest crowns per hectare (Figure 4A,B). Additionally, the predicted canopy tree density from each algorithm was compared to the photo‐interpreted inventory (Figure 4C). Overall, the ITC models reproduced field values with varying accuracy depending on the algorithm. For the median area of the 20 largest crowns per hectare (Figure 4A), Detectree2SAM achieved the lowest prediction error (RMSD = 43.77 m^2^–26%), followed by SAM (52.85 m^2^–31.5%) and Detectree2 (89.31 m^2^–53.1%). Regarding the total area (Figure 4B), Detectree2SAM again showed the best performance (RMSD = 1235.2 m^2^–28.9%), ahead of SAM (1310.48 m^2^–30.6%) and Detectree2 (1670.05 m^2^–39%). In terms of canopy tree density (Figure 4C), SAM provided the most accurate predictions (RMSD = 19.7 ind/ha–22.2%), whereas Detectree2SAM tended to strongly overestimate densities (RMSD = 56 ind/ha–63.2%).

Following linear correction, prediction accuracy improved across all algorithms and metrics (Table 2, Figure 4). Reductions in RMSD ranged from 5.7% (SAM, density) to 75.3% (Detectree2SAM, median crown area), with the largest gains observed for crown area metrics. The relative ranking of algorithms was preserved after correction: Detectree2SAM showed the lowest RMSD for both crown area metrics, while SAM showed the lowest RMSD for canopy density.

**TABLE 2:** Linear correction equations and their effect on prediction accuracy for each algorithm and canopy metric at plot scale.

Metric	Method	Correction equation	RMSD (original)	RMSD (corrected)	RMSD reduction (%)
Median Area (m^2^)	Detectree2	*y* = **63.42** + **0.43**·*x*	89.31	28.01	**68.6**
	SAM	*y* = **51.69** + **0.56**·*x*	52.85	22.17	**58.1**
	Detectree2SAM	*y* = **8.46** + **0.76**·*x*	43.77	10.81	**75.3**
Sum Area (m^2^)	Detectree2	*y* = **2147.50** + **0.38**·*x*	1670.05	560.39	**66.4**
	SAM	*y* = **1192.03** + **0.57**·*x*	1310.48	477.51	**63.6**
	Detectree2SAM	*y* = **1518.90** + **0.52**·*x*	1235.20	502.28	**59.3**
Density (ind/ha)	Detectree2	*y* = **−45.75** + **1.10**·*x*	38.59	19.63	**49.1**
	SAM	*y* = **17.40** + **0.86**·*x*	19.67	18.55	**5.7**
	Detectree2SAM	*y* = **−17.37** + **0.75**·*x*	55.99	18.53	**66.9**

*Note:* For each combination of algorithm and metric, the correction equation (*y* = *a* + *b*·*x*) was derived from the regression of observed against predicted values (Observed ~ Predicted). RMSD (original) and RMSD (corrected) are expressed in native units (m^2^ for crown area metrics, ind/ha for density). RMSD reduction (%) represents the relative gain in prediction accuracy following correction. Bold values indicate mean performance estimates; values in parentheses indicate 95% bootstrap confidence intervals.

### 3.3 Spatial Comparison of Plot‐Level Canopy Structure Attributes at the Landscape Scale

**FIGURE 5:**
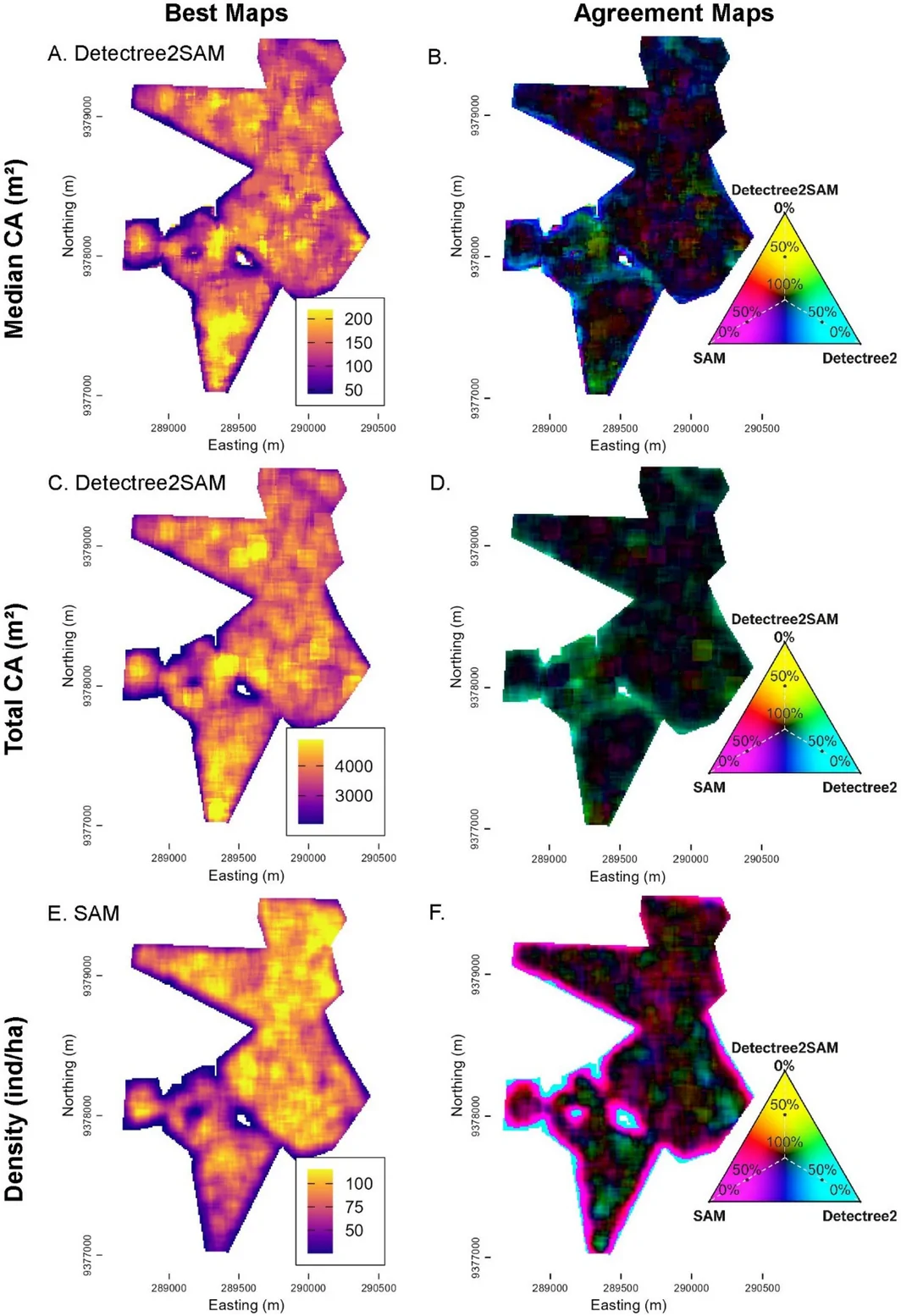
Maps of linearly corrected canopy structural attributes derived from the best‐performing algorithm for each metric (left column) and inter‐algorithm agreement maps based on corrected values (right column). Values were computed using a 10 m × 10 m moving window with 1 ha aggregation, and linearly corrected to reduce systematic bias identified at the plot scale. In the agreement maps (B, D, F), hue indicates the relative contribution of each algorithm (cyan = Detectree2; magenta = SAM; yellow = Detectree2SAM; intermediate hues reflect mixed contributions), while brightness reflects the magnitude of local deviation from the cross‐algorithm mean: Lighter pixels indicate stronger disagreement, darker pixels indicate higher agreement. (A) Median crown area of the 20 largest crowns per hectare (m^2^)—Detectree2SAM. (B) Inter‐algorithm agreement for median crown area. (C) Total crown area of the 20 largest crowns per hectare (m^2^)—Detectree2SAM. (D) Inter‐algorithm agreement for total crown area. (E) Canopy tree density (ind/ha)—SAM. (F) Inter‐algorithm agreement for canopy tree density. Inset triangles show the relative dominance of each algorithm across all pixels for each metric.

Continuous mapping of plot‐level derived parameters was performed for the entire Luki study site. The maps were generated using linearly corrected values (Table 2) from the algorithms that showed the closest agreement with the reference dataset according to results presented in Figure 4. The maps of the median and sum of the 20 largest crowns per hectare were produced from the Detectree2SAM outputs (Figure 5A–C), while the canopy crown density map was generated from the SAM outputs (Figure 5E).

The map of median areas of the 20 largest crowns per hectare (Figure 5A) highlights localized zones with large tree crowns, potentially indicative of stands dominated by large trees—this metric could thus serve as a proxy for dominant height. Simultaneously, the map of total areas of the 20 largest crowns per hectare (Figure 5C) highlights zones where dominant trees tend to have larger stem diameters, reflecting the allometric relationship between crown area and DBH. Finally, the map of tree density per hectare (Figure 5E) reveals a heterogeneous spatial distribution of canopy density across the study area, with notable variation among plots. This representation highlights strong local contrasts often masked by plot‐level averages.

The agreement maps for the median crown area (Figure 5B) show generally dark tones across most of the study site, indicating an overall good convergence between the three algorithms. However, several spatial patterns of disagreement are visible. A clear border effect appears along the edges and canopy gaps, dominated by blue tones, indicating that Detectree2 and SAM jointly deviate from the cross‐algorithm mean in these transitional areas—contexts typically associated with regeneration or disturbed stands where crown boundaries are smaller and more irregular. Purple tones frequently observed across the interior suggest that SAM and Detectree2 jointly diverge from the cross‐algorithm mean, potentially reflecting zones of closed canopy where delimiting adjacent crowns remains ambiguous. Scattered green patches indicate localized areas where Detectree2 and Detectree2SAM deviate together from the cross‐algorithm mean.

For the sum of the areas of the 20 largest crowns per hectare (Figure 5D), a generally strong agreement between algorithms is also observed. However, green tones dominate in many areas, indicating that Detectree2 and Detectree2SAM jointly deviate upward from the cross‐algorithm mean—a mean that is itself pulled downward by SAM's tendency to under‐segment the canopy. Scattered purple patches reflect localized areas where Detectree2 and SAM jointly deviate from this mean.

Finally, for canopy crown density (Figure 5F), magenta tones dominate across large portions of the site, reflecting SAM's systematic tendency to underestimate crown density. Border effects are clearly visible as bright magenta and cyan fringes along the edges of the study area, representing methodological artifacts that could be particularly problematic for analyses of forest fragmentation or edge effects.

### 3.4 Comparison of Individual and Plot‐Level Attributes at the Landscape Scale

**FIGURE 6:**
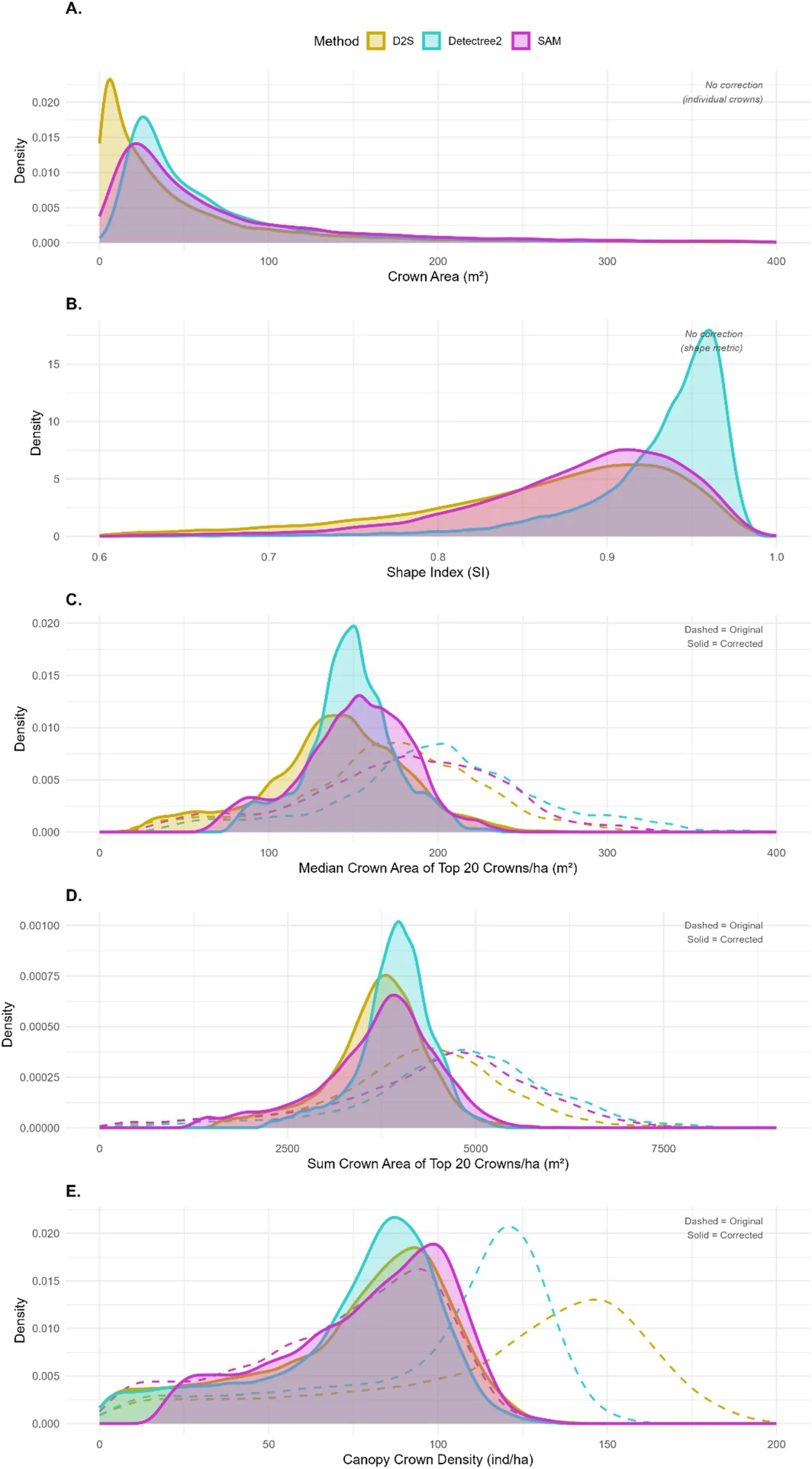
Distribution of forest canopy structural attributes in the Luki Biosphere Reserve, comparing tree‐level and plot‐level estimates obtained from three automated crown delineation methods (Detectree2, SAM, and Detectree2SAM). The density plots illustrate the probability distributions of each attribute for the three methods, enabling direct comparison of their structural estimates across the Luki landscape. (A) Distribution of individual canopy crown areas (m^2^). (B) Distribution of individual crown shape index (SI). (C–E) For plot‐level metrics, dashed lines show the original distributions and solid filled curves show the linearly corrected distributions. (C) Distribution of plot‐level median area of the 20 largest crowns per hectare (m^2^). (D) Distribution of plot‐level total area of the 20 largest crowns per hectare (m^2^). (E) Distribution of plot‐level canopy crown density (ind/ha).

The continuous distribution of crown areas for the three segmentation algorithms across the entire study site reveals marked differences (Figure 6A; Table S1). Detectree2 is slightly shifted toward larger crowns (median area: 45.1 m^2^; SD: 94.2 m^2^). SAM displays a broader distribution, capturing a wide range of crown sizes (median area: 44.7 m^2^; SD: 107.5 m^2^). Detectree2SAM shows the highest density of small crowns, mostly below 50 m^2^, reflecting a more conservative segmentation (median area: 26.3 m^2^; SD: 83.4 m^2^).

Crown shape indices also reveal notable differences among the three approaches (Figure 6B). Detectree2 shows a strong density around indices close to 1, suggesting very regular and often circular crowns (median SI: 0.94; SD: 0.05). SAM exhibits high density for indices below 0.8, indicating more complex crown shapes (median SI: 0.90; SD: 0.07). Detectree2SAM shows a distribution similar to SAM but slightly more spread out, with indices mostly between 0.7 and 1, suggesting greater variability in the crown shapes detected (median SI: 0.88; SD: 0.08).

The median areas of the 20 largest crowns per plot show little difference among the three methods before correction (Figure 6C). Detectree2SAM shows the lowest spread (median: 173.9 m^2^; SD: 55.3 m^2^), Detectree2 the highest median values (199.1 m^2^; SD: 63.2 m^2^), and SAM falls in between (182.3 m^2^; SD: 59.5 m^2^). These differences are attenuated by the selection constraint, which limits the analysis to the 20 largest trees per hectare. Following correction, all three distributions shift toward lower values: Detectree2SAM (141.3 m^2^; SD: 42.2 m^2^), Detectree2 (148.7 m^2^; SD: 27.0 m^2^), and SAM (153.4 m^2^; SD: 33.2 m^2^).

The sum of the areas of the 20 largest crowns per plot follows a similar pattern (Figure 6D). Detectree2SAM has the lowest total area and the most homogeneous spread (median sum: 4296.8 m^2^; SD: 1276 m^2^). Detectree2 tends toward higher values (4826.8 m^2^; SD: 1301.5 m^2^), while SAM shows greater heterogeneity (4623.4 m^2^; SD: 1400.2 m^2^). After correction, all distributions shift toward lower values: Detectree2SAM (3759.2 m^2^; SD: 665.3 m^2^), SAM (3823.6 m^2^; SD: 796.9 m^2^), and Detectree2 (3971.2 m^2^; SD: 491.7 m^2^).

Canopy tree density reveals more pronounced differences among the algorithms (Figure 6E). SAM is centered on the lowest densities, with a peak between 70 and 110 trees per hectare, indicating a tendency to detect fewer trees per plot (median density: 78 ind/ha; SD: 29.3 ind/ha). Detectree2 shows an intermediate distribution, with a peak between 110 and 130 trees per hectare (median density: 112 ind/ha; SD: 35.9 ind/ha). Detectree2SAM is clearly shifted toward the highest densities (120–180 ind/ha), indicating a much larger number of detected trees (median density: 130 ind/ha; SD: 45.1 ind/ha). Following correction, all three algorithms converge toward lower density values: Detectree2 (77.8 ind/ha; SD: 39.6 ind/ha), Detectree2SAM (80.3 ind/ha; SD: 33.9 ind/ha), and SAM (84.5 ind/ha; SD: 25.2 ind/ha).

## 4 Discussion

In this study, we showed that Detectree2 and Detectree2SAM detect more crowns but with more segmentation errors (false positives or over‐segmentation), whereas SAM produces more conservative results with fewer detections but higher precision (Table 1). Overall, the performances we obtained are comparable to or slightly better than those reported in other tropical and subtropical forests: Ball et al. (2023) reported an IoU‐based F1 of 0.64 for Detectree2 in Malaysian Borneo and French Guiana, rising to 0.74 for the tallest trees—consistent with our own scores of 0.63 (Detectree2) and 0.67 (SAM). In zero‐shot segmentation benchmarks across NEON and Detectree2 datasets, Chen et al. (2025) reported precision and recall values of 0.17–0.61 and 0.41–0.76 respectively for DeepForest, Detectree2, and SAM2, highlighting the wide variability in performance across methods and contexts. Studies validating these models against terrestrial LiDAR (TLS) reference data tend to report substantially lower scores—Allen et al. (2025) obtained F1 values of 0.23–0.31 for Detectree2 when validated against TLS in closed‐canopy forests, compared to 0.67 when validated against manual labels at the same sites—likely because TLS provides a higher spatial precision that exposes segmentation errors not detectable with photointerpretation‐based reference datasets. In other contexts, such as open‐canopy forests in the National Ecological Observation Network, DeepForest achieved a recall of 0.69 and a precision of 0.61 (Weinstein et al. 2019), suggesting that the results obtained here in structurally more complex tropical forests are broadly comparable to those achieved in less dense environments.

Detectree2‐based methods tend to detect more crowns while maintaining a reasonable balance between recall and precision. However, direct comparisons across studies remain challenging given the strong influence of site‐specific conditions. Notable variations in canopy structure and tree density can affect detection efficiency depending on the study site (Weinstein et al. 2020).

Moreover, forest cover composition—whether monospecific or mixed‐species—influences canopy structure and crown morphology (Pretzsch 2014), which in turn can affect the performance of automated segmentation methods. The spatial arrangement of trees, their density, as well as the degree of crown overlap are also key factors influencing detection efficiency (Gu et al. 2020). Finally, sensor characteristics (e.g., spatial, spectral, and radiometric resolution) and acquisition conditions (e.g., sun‐scene‐sensor geometries) also influence the quality of the results. For example, Gan et al. (2023) showed that the performance of the pre‐trained Detectree2 model decreases as image resolution becomes coarser, with both precision and recall being negatively affected. Added to this is the heterogeneity in model calibrations, often trained on data from very different sensors and environmental contexts—or, as with SAM, without initial calibration for forest applications, which can lead to inconsistent performance across studies. Moreover, validation strongly depends on the reference method used. TLS, although more precise and restrictive (Allen et al. 2025), appears to be costly, complex to process for reliable results, and sensitive to occlusion, as shown in Cameroon (Momo Takoudjou et al. 2018). Conversely, photointerpretation, being more accessible, allows for the generation of a larger number of observations, albeit at the cost of potentially lower accuracy (Bastin et al. 2017). Here, our approach, based on synchronized field digitization, offers a compromise: it improves the representativeness of the observations vs. TLS, better suited to the ecological specificities of the study site, while guaranteeing a high level of quality due to the synchronicity in field‐aerial acquisition.

Although F1‐scores and recall values are key indicators of overall detection performance, our results show that the choice of segmentation model can also lead to overall over‐ or under‐segmentation. This highlights that these indicators remain insufficient to fully evaluate the quality of crown segmentation necessary to understand the distribution of tree size and shape within a given forested landscape (Ball et al. 2023; Li et al. 2012; Stewart et al. 2021; Weinstein et al. 2020). Yet, studying the distribution of tree crowns over a given area remains crucial to understand key ecological differences within and between forest landscapes. At the individual tree scale, reproducing crown morphology through shape and area metrics informs architectural traits. At the landscape scale, the derived metric of largest crown area and density provide essential but underexplored insights into forest structure—for example, on forest dynamics or stage of succession (Matsuo et al. 2021). Moreover, in many studies, ITC delineation serves as a baseline for long‐term monitoring of individual crown changes over time—including phenological cycles (Kleinsmann et al. 2023; Park et al. 2019) or tree mortality (Allen et al. 2024; Yao et al. 2024).

Detectree2 shows the highest completeness, that is, the percentage of observed trees detected, with a superior recall, leading to more credible estimates of canopy tree numbers. Although SAM provides a number of individuals close to that of photo‐interpretation, ITC methods, whether manual or automatic, tend to underestimate this number, notably due to the under‐detection of small crowns (Ball et al. 2023) and crown fusion (Feret and Asner 2013). This completeness is important because individual tree detection allows for the analysis of growth, mortality, and recruitment—fundamental processes for understanding forest demographic dynamics (Leite et al. 2024). These data are also essential for estimating the dynamic equilibrium of forest populations (Farrior et al. 2016; Muller‐Landau et al. 2006; West et al. 2009). Furthermore, the number of detected trees serves as a proxy for forest cover, indicating ecosystem health and productivity, influencing light interception, microclimate, and habitat availability for many species (Lines et al. 2022). Detectree2 also tends to detect larger crowns than other methods and tends to oversimplify crown shapes (regardless of the smoothing parameters used—Figure S4).

With its generalist parameters, SAM exhibits a trade‐off between high precision and reduced recall, remaining very conservative in the number of detected trees. This approach minimizes false positives but leads to under‐detection of crowns. Nevertheless, it captures a broader range of crown shape complexity than Detectree2, suggesting interesting potential for automated detection of canopy trees architectural properties, as indicated by the distribution of shape indices. Understanding the complexity of crown forms can help infer certain physiological strategies—particularly shade or heat tolerance (Arseniou and MacFarlane 2021; Echereme et al. 2015)—as well as other functional strategies. Indeed, competition for light induces specific morphological responses such as the development of narrow or shallow crowns, or greater investment in height growth (Jucker et al. 2017), reflecting adaptive trade‐offs to local environmental conditions. It also helps to highlight phenomena like crown abrasion, which leads to crown shyness (Hajek et al. 2015). Beyond vertical structure, the shape and space an individual tree is able to occupy dynamically—that is, its plasticity—plays a central role in canopy space use (Jucker et al. 2015). Understanding canopy structure and complexity through fine‐scale segmentation provides valuable indicators of forest productivity, biodiversity, and ecosystem responses to environmental change (Lines et al. 2022). It should also be noted that the precision–recall balance reported here partly depends on post‐processing filtering parameters, including the acceptance threshold and minimum crown size. In this study, we adopted the default parameters from the original implementations (Ball et al. 2023; Kirillov et al. 2023) throughout, rather than tuning them per site: this keeps the comparison between algorithms neutral, keeps the pipeline reproducible on other sites without expert intervention, and—most importantly—matches the logic of the correction framework itself, which assumes systematic biases and corrects for them at the plot scale rather than trying to eliminate them upstream through manual tuning. The under‐detection we observe for SAM under these default settings is consistent with recent reports of out‐of‐the‐box under‐segmentation in forest canopies (Chen et al. 2025; Teng et al. 2025) and is exactly the type of systematic bias the correction step is designed to absorb. Future work could systematically explore how site‐specific fine‐tuning of model weights and hyperparameters influences segmentation performance and the resulting precision–recall trade‐offs across different forest types and objectives.

Detectree2SAM detects a higher number of trees per hectare compared to Detectree2 and SAM. However, this increase partly results from over‐segmentation, linked to SAM's ability to generate a mask containing multiple spatially disjoint objects in response to an ambiguous centroid. During post‐processing, these multiple components are extracted as distinct segments, leading to a number of segments exceeding the initial number of centroids, as well as a higher number of false positives, as confirmed by IoU‐based validation. Nevertheless, the distribution of shape indices shows that Detectree2SAM better captures crown complexity compared to Detectree2, reflecting a more faithful representation of trees' architectural diversity. Detectree2SAM thus positions itself as a compromise between the crown over‐simplification produced by Detectree2 and the under‐segmentation observed with SAM, offering a better representation of morphological complexity while maintaining a high detection capacity (Figure 6B). It also provides the best match with field data for representing large canopy trees at plot scale, highlighting its robustness in characterizing dominant structures and a diversity of functional strategies.

Beyond these three methods, new generalist and tree‐specific segmentation models are emerging rapidly, such as SAM2 or TreePseCo, which could be included in future comparisons to assess robustness across forest types (Chen et al. 2025; Lungo Vaschetti et al. 2025).

### 4.1 Promoting Landscape‐Scale Analysis Through Largest Trees

Our results show that overall crown size distributions differ substantially depending on the algorithm used, which complicates their ecological interpretation. In contrast, when focusing on the 20 largest crowns per hectare, we observe a much better convergence between methods, reflecting a higher reliability of detection for these largest trees (Figure 6C,D), which are generally better segmented. This finding highlights the value of focusing future methodological approaches on this subset of trees to achieve a more robust characterization of forest structural attributes at the landscape scale when using automatic segmentation algorithms. Importantly, the raw structural metrics produced by all three algorithms exhibit systematic biases, making their direct ecological interpretation unreliable. Consequently, the linear correction derived from plot‐scale field observations should be considered a necessary step rather than an optional refinement for quantitative applications of ITC‐derived products. Before correction, RMSD values ranged from 26% to 53%. Although substantial errors remain after correction, this does not preclude the use of these data for structural monitoring, provided that residual errors are approximately unbiased. Under this condition, positive and negative residuals tend to compensate when aggregated over larger spatial units, reducing their influence on landscape‐scale structural metrics. This principle has also been illustrated in large‐scale remote sensing applications, where products with substantial local uncertainty can still provide reliable estimates after spatial aggregation (Xu et al. 2017). Accordingly, the framework proposed here should be viewed primarily as a tool for structural characterization and relative monitoring rather than for absolute carbon stock estimation or precise tree counting. The spatial transferability of the correction factors nonetheless remains an open question, as they were calibrated at a single site; users extending this approach to new sites should validate them against local field data before landscape‐scale application. Subject to this caveat, this approach may help harmonize forest structural assessments across datasets derived from different acquisition conditions and processing workflows (Barbier and Couteron 2015).

Analyzing all crowns across the study area reveals substantial variability in results between algorithms ranging from 26.3 to 45.1 m^2^ (Figure 6A; Table S1). For comparison, Blanchard et al. (2016), based on ground measurements of crown dimensions, reported median crown areas of 50 m^2^ for the Cameroon–Gabon region, 31 m^2^ for Indonesia, and 25 m^2^ for India. The values obtained in this study fall within this range, though such comparisons across studies and ecosystems should be interpreted with caution, as observed differences may reflect a combination of site‐specific environmental factors, algorithmic tendencies, and methodological choices rather than a true ecological signal. The value obtained for Detectree2SAM, which is closer to those reported for Asian sites, may be explained by its tendency toward over‐segmentation, whereas the values from Detectree2 and SAM suggest slightly larger crowns than those typically reported for Central African tropical forests. These differences may result from site‐specific environmental factors, such as low rainfall influencing maximum tree height (Gorel et al. 2025), topography affecting drainage and exposure (Shenkin et al. 2020), or tree social status (dominant vs. subordinate) which determines the proportion of crown surface exposed to the canopy. However, these effects are generally less pronounced on crowns than on stems, which are more sensitive to soil and slope variations (Antin et al. 2013).

Crown density per hectare also shows marked differences between models, both spatially and descriptively. More broadly, the differences observed between models—whether in crown area, shape indices, or densities—may also stem from acquisition biases, intrinsic tendencies of each algorithm (e.g., over‐segmentation), methodological choices such as hyperparameters or post‐processing steps, as well as the potential influence of study area size on the sensitivity of derived structural metrics (Jiménez 2000; Saatchi et al. 2011).

Furthermore, the apparent convergence observed when aggregating metrics at plot scale should be interpreted with caution: by averaging individual crown metrics over a fixed spatial unit, plot‐scale estimates mechanically reduce variance and can strengthen variable relationships independently of any true ecological signal (aggregation bias—Jelinski and Wu 1996). This property, however, is precisely what makes the landscape‐scale aggregation approach operationally robust: it yields more stable structural estimates across algorithms, regardless of their individual segmentation tendencies (Chave et al. 2004; Clark and Clark 2000). Applying linear correction factors derived from plot‐scale regressions further reinforced this stability by mitigating systematic prediction biases, with several metrics shifting toward lower values and showing greater convergence after correction—suggesting that part of the observed variability originated from systematic bias rather than true ecological variability (Figure 6). This convergence is particularly pronounced when focusing on the 20 largest trees per hectare, as confirmed by the agreement maps for median and sum crown areas (Figure 5). Beyond algorithmic comparisons, this approach also enables ecologically meaningful spatial patterns to be highlighted, such as the marked presence of large trees in the southern part of the site, characterized by a former plantation area where *Terminalia superba* Engl. & Diels has been favored.

### 4.2 Perspectives

Building on these findings, our results show that adopting a landscape‐scale perspective built from a plot‐level characterization of forest attributes provides a consistent framework, regardless of the ITC algorithm chosen, for further investigating complex ecological phenomena within tropical forests. Beyond crown size distributions and largest tree structure, this approach opens avenues for exploring forest demography and physiological strategies, as discussed earlier. It is particularly suited for studying canopy gap size and distribution—key drivers of forest regeneration dynamics (Goulamoussène et al. 2017)—as well as monitoring phenological changes that are sensitive to climate change (Abernethy et al. 2018; Bohlman 2024). The systematic biases observed here may partly stem from a mismatch between algorithm training contexts and Central African forest conditions, raising the question of whether similar correction factors would apply across the region—and whether biases are primarily driven by training data characteristics or local structural specificities.

This perspective further allows for the assessment of anthropogenic impacts such as logging and habitat fragmentation by integrating disturbance history (Arroyo‐Rodríguez et al. 2017; Asner et al. 2009; Dupuis et al. 2023). Working at this scale strengthens the robustness of structural analyses by accounting for variability in forest density, architecture, and topography. Finally, it supports the identification of the most influential large‐scale drivers through the use of “super‐sites,” paving the way for regional and even global model calibrations and algorithm adjustments (Chave et al. 2019; Labrière et al. 2018).

## 5 Conclusion

Our study demonstrates that ITC segmentation algorithms applied to low‐cost UAV imagery produce substantial, algorithm‐specific systematic biases in structural metrics, and that these biases must be explicitly corrected before ecological interpretation. We propose and validate an operational pipeline combining (i) plot‐scale field validation of structural metrics, (ii) linear bias correction calibrated per algorithm, and (iii) aggregated landscape‐scale analysis. Applied to the Luki Biosphere Reserve (DRC), this pipeline substantially reduces interalgorithm divergence and yields consistent structural estimates at landscape scale.

We show that focusing on the 20 largest crowns per hectare improves cross‐algorithm consistency and ecological interpretability, and that a simple linear correction framework derived from plot‐scale field data substantially reduces systematic prediction biases. Together, these two elements provide a practical and transferable approach for multi‐scale forest structural monitoring using affordable UAV imagery.

Beyond the methodological contribution, this framework opens concrete perspectives for ecologically relevant applications—including forest demographic monitoring, canopy gap dynamics, phenological change detection, and the assessment of anthropogenic disturbance—at spatial scales previously inaccessible through field inventories alone. Ultimately, it contributes to bridging the gap between detailed but spatially restricted ground data and broad‐scale remote sensing products, supporting repeated and harmonized assessments of tropical forest state.

## Author Contributions

**Antoine Plumacker:** conceptualization (equal), data curation (equal), formal analysis (equal), investigation (equal), methodology (equal), software (equal), validation (equal), visualization (equal), writing – original draft (equal), writing – review and editing (equal). **Bhely Angoboy Ilondea:** funding acquisition (equal), resources (equal), writing – review and editing (equal). **Nicolas Barbier:** resources (equal), writing – review and editing (equal). **Thibauld Collet:** writing – review and editing (equal). **Hugo de Lame:** writing – review and editing (equal). **Pauline Depoortere:** writing – review and editing (equal). **Alexia Favaro:** writing – review and editing (equal). **Adeline Fayolle:** funding acquisition (lead), project administration (equal), resources (equal), writing – review and editing (equal). **Marjane Kaddouri:** writing – review and editing (equal). **Carmel Mounziegou‐Mounziegou:** validation (equal), writing – review and editing (equal). **Pierre Ploton:** methodology (equal), resources (equal), writing – review and editing (equal). **Zoé Rousseau:** writing – review and editing (equal). **Edmond Saint Denis:** software (equal). **Paul Tresson:** writing – review and editing (equal). **Hadrien Tulet:** validation (equal), writing – review and editing (equal). **Arthur Vander Linden:** methodology (equal), software (equal), writing – review and editing (equal). **Jean‐François Bastin:** conceptualization (equal), formal analysis (equal), funding acquisition (equal), investigation (equal), investigation (equal), methodology (equal), methodology (equal), project administration (equal), project administration (equal), resources (equal), resources (equal), supervision (lead), supervision (lead), visualization (equal), visualization (equal), writing – original draft (equal), writing – original draft (equal), writing – review and editing (equal), writing – review and editing (equal).

## Funding

This work was supported by EOS Canopi (O.0026.22).

## Conflicts of Interest

The authors declare no conflicts of interest.

## Supporting information


**Figure S1:** Field‐based crown delineation workflow using QField. (a) Overview of the drone orthomosaic with pre‐delineated tree crowns (green polygons) displayed on a tablet in the field. (b) Field team navigating to a target tree while consulting the orthomosaic on a tablet. (c) Close‐up view of a target tree crown prior to field verification. (d) Manual refinement of the crown boundary directly on the orthomosaic using QField, with the edited polygon shown in red.
**Figure S2:** Distribution of Intersection over Union (IoU) values between the field‐delineated crowns and their best‐matching photo‐interpreted crown across plots. IoU was computed for each field‐delineated crown as the ratio of the intersection area to the union area with the most overlapping photo‐interpreted crown.
**Figure S3:** Illustration of the benefit of multi‐date photointerpretation for individual tree crown delineation in the Luki Biosphere Reserve. The same crowns (orange polygons) are shown on two orthomosaics acquired at different dates: (a) 04 May 2023, where the dense and homogeneous canopy makes crown boundaries difficult to distinguish, and (b) 01 November 2023, where contrasting colorimetry driven by differential phenological activity and changes in acquisition conditions reveals canopy structure more clearly, facilitating crown boundary delineation.
**Figure S4:** Effect of the smoothing parameter in Detectree2 post‐processing on crown geometry. Three individual crowns are shown at three smoothing levels: 0 (no smoothing, retaining pixel‐level noise artifacts), 0.1 (low smoothing), and the value used in this study. The figure illustrates that the smoothing step reduces geometric noise at crown boundaries without substantially altering the overall crown shape or shape index.
**Figure S5:** Example of shape index (circularity index) derived from segments produced by the Detectree2SAM ITC model, ranging from the simplest to the most complex shape.
**Table S1:** Median and standard deviation for each metric and model.

## Data Availability

All data needed to evaluate the conclusions in the paper are present in the paper, in the Supporting Information, and on the Github repository: https://github.com/APlumacker/Detectree2SAM.
