# Supplementary material for: Toward Consistent Canopy Characterization From Low‐Cost UAV Imagery
Source: Ecol Evol. 2026 Sep 2;16(9):e74260. doi: 10.1002/ece3.74260 (PMC13539220; doi:10.1002/ece3.74260)
Supplement: Supplementary file 1 [file ECE3-16-e74260-s001.docx]

**Appendix - Monitoring plot-level tropical forest canopy structure with automated crown segmentation from low-cost drone imagery**

**Appendix**

**Supp. Fig. 1 Field-based crown delineation workflow using QField. (a) Overview of the drone orthomosaic with pre-delineated tree crowns (green polygons) displayed on a tablet in the field. (b) Field team navigating to a target tree while consulting the orthomosaic on a tablet. (c) Close-up view of a target tree crown prior to field verification. (d) Manual refinement of the crown boundary directly on the orthomosaic using QField, with the edited polygon shown in red.**

**Supp. Fig. 2. Distribution of Intersection over Union (IoU) values between the field-delineated crowns and their best-matching photo-interpreted crown across plots. IoU was computed for each field-delineated crown as the ratio of the intersection area to the union area with the most overlapping photo-interpreted crown.**

**Supp. Fig. 3. Illustration of the benefit of multi-date photointerpretation for individual tree crown delineation in the Luki Biosphere Reserve. The same crowns (orange polygons) are shown on two orthomosaics acquired at different dates: (a) 04 May 2023, where the dense and homogeneous canopy makes crown boundaries difficult to distinguish, and (b) 01 November 2023, where contrasting colorimetry driven by differential phenological activity and changes in acquisition conditions reveals canopy structure more clearly, facilitating crown boundary delineation.**

**Supp. Fig. 4. Effect of the smoothing parameter in Detectree2 post-processing on crown geometry. Three individual crowns are shown at three smoothing levels: 0 (no smoothing, retaining pixel-level noise artifacts), 0.1 (low smoothing), and the value used in this study. The figure illustrates that the smoothing step reduces geometric noise at crown boundaries without substantially altering the overall crown shape or shape index.**

**Supp. Fig. 5. Example of shape index (circularity index) derived from segments produced by the Detectree2SAM ITC model, ranging from the simplest to the most complex shape.**

| **Supp. Table. 1. Median and standard deviation for each metric and model** | | | |
| --- | --- | --- | --- |
| **Metric** | **Model** | **Median** | **SD** |
| **Crown Area (m²)** | D2S | 26.27 | 83.38 |
|  | Detectree2 | 45.07 | 94.19 |
|  | SAM | 44.67 | 107.53 |
| **Shape Index (SI)** | D2S | 0.88 | 0.08 |
|  | Detectree2 | 0.94 | 0.05 |
|  | SAM | 0.90 | 0.07 |
| **Median Crown Area (Top 20)** | D2S | 173.87 | 55.28 |
|  | Detectree2 | 199.14 | 63.20 |
|  | SAM | 182.35 | 59.52 |
| **Sum Crown Area (Top 20)** | D2S | 4296.77 | 1275.99 |
|  | Detectree2 | 4826.78 | 1301.45 |
|  | SAM | 4623.39 | 1400.16 |
| **Canopy Crown Density (ind/ha)** | D2S | 130.00 | 45.13 |
|  | Detectree2 | 112.00 | 35.86 |
|  | SAM | 78.00 | 29.27 |
